# TTF-1与小细胞肺癌一线化疗敏感性及预后的相关性分析

**DOI:** 10.3779/j.issn.1009-3419.2020.101.27

**Published:** 2020-07-20

**Authors:** 冰睿 王, 暖 丰, 新艳 石, 琦 綦, 晓蕊 迟, 婷婷 宋, 红梅 李

**Affiliations:** 1 266003 青岛，青岛大学附属医院肿瘤内科 Department of Oncology, The Affiliated Hospital of Qingdao University, Qingdao 266003, China; 2 266000 青岛，青岛市妇女儿童医院营养科 Department of Nutriology, Qingdao Women and Children's Hospital, Qingdao 266000, China

**Keywords:** 肺肿瘤, TTF-1, 化疗敏感性, 预后, 生物标志物, Lung neoplasms, TTF-1, Chemotherapy sensitivity, Prognosis, Biomarker

## Abstract

**背景与目的:**

甲状腺转录因子-1（thyroid transcription factor-1, TTF-1）在非小细胞肺癌中被广泛研究，被认为是非小细胞肺癌的独立预后因子，但TTF-1在小细胞肺癌（small cell lung cancer, SCLC）中的预后价值研究较少。本研究旨在探讨TTF-1的表达状态与SCLC患者一线化疗敏感性及预后的关系。

**方法:**

回顾性分析2017年1月1日-2019年1月1日在青岛大学附属医院确诊并治疗的、一线应用以铂类为基础的化疗的SCLC患者234例，随访患者临床特征及治疗、生存情况，采用*χ*^2^检验及*Logistic*回归模型分析TTF-1的表达状况与化疗反应率的关系，*Kaplan-Meier*法和*Cox*比例风险回归模型分析TTF-1的表达对患者生存期的影响。

**结果:**

234例患者中，TTF-1阳性表达188例（80.3%, 188/234），TTF-1阴性表达46例（19.7%, 46/234），TTF-1阳性患者一线化疗客观反应率（objective response rate, ORR）高于阴性患者（70.7% *vs* 47.8%）（*χ*^2^=8.681, *P*=0.003）。*Logistic*回归多因素分析显示，TTF-1的表达是一线化疗ORR的独立预测因素（OR=0.216, 95%CI: 0.076-0.615, *P*=0.004），但此差异仅体现在局限期SCLC（limited-stage SCLC, LS-SCLC）中。TTF-1阴性表达患者中位无进展生存期（progression free survival, PFS）6.9个月短于TTF-1阳性表达患者的9.0个月（*χ*^2^=9.357, *P*=0.002）。TTF-1阴性组患者中位总生存期（overall survival, OS）13.3个月短于TTF-1阳性组患者的20.1个月（*χ*^2^=12.082, *P*=0.001）。

**结论:**

TTF-1表达状态为SCLC患者一线化疗反应率及生存的独立预测因素，可能成为预测SCLC治疗疗效及预后的生物标志物。

## Abstract (en)

**Background and objective:**

Thyroid transcription factor-1 (TTF-1) has been widely studied in non-small cell lung cancer, which is considered as an independent prognostic factor in patiens with non-small cell lung cancer. However, there are few studies on the prognostic value of TTF-1 in small cell lung cancer (SCLC). The purpose of this study was to explore the relationship between the expression state of TTF-1 and the sensitivity to first-line chemotherapy and prognosis in patients with SCLC.

**Methods:**

A retrospective analysis was made on 234 patients with SCLC who were diagnosed and treated in The Affiliated Hospital of Qingdao University and received platinum-based chemotherapy. The clinical characteristics, treatment and survival of the patients were followed up. Chi *χ*^2^ test and *Logistic* regression model were used to analyze the relationship between TTF-1 expression and chemotherapy response rate. *Kaplan-Meier* method and *Cox* proportional hazard regression model were used to analyze the effect of TTF-1 expression on survival time of patients.

**Results:**

Among the 234 patients, the positive expression of TTF-1 was 188 cases (80.3%), and the negative expression of TTF-1 was 46 cases (19.7%). The objective response rate (ORR) of first-line chemotherapy in patients with positive expression of TTF-1 was higher than that in patients with negative expression of TTF-1 (70.7% *vs* 47.8%) (*χ*^2^=8.681, *P*=0.003). *Logistic* regression multivariate analysis showed that the expression state of TTF-1 was an independent predictor of ORR in first-line chemotherapy (OR=0.216, 95%CI: 0.076-0.615, *P*=0.004), however this difference was only reflected in LS-SCLC. The median progression free survival (PFS) of patients with negative expression of TTF-1 was shorter than that of patients with positive expression (6.9 months *vs* 9.0 months) (*χ*^2^=9.357, *P*=0.002). The median OS in TTF-1 negative group was shorter than that in TTF-1 positive group (13.3 months *vs* 20.1 months)(*χ*^2^=12.082, *P*=0.001).

**Conclusion:**

TTF-1 expression is an independent predictor of first-line chemotherapy response rate and survival in patients with SCLC, and may become a biomarker to predict the efficacy and prognosis of SCLC.

小细胞肺癌（small cell lung cancer, SCLC）是一种高度恶性的肺癌亚型，占所有肺癌病理类型的10%-15%^[1]^。虽然SCLC最初对细胞毒药物和放射治疗敏感，但绝大多数病例会复发并表现出耐药性。在过去的几十年里，其治疗进展不大，患者的预后仍然很差。甲状腺转录因子-1（thyroid transcription factor-1, *TTF-1*）是*NKx2*转录基因家族成员之一，在维持肺正常的结构与功能中起重要作用，是肺上皮细胞分化和肺形态发生的主要调节因子，其表达在SCLC中明显上调^[2]^。研究^[3-7]^显示，TTF-1阳性表达与表皮生长因子受体（epidermal growth factor receptor, *EGFR*）高突变率相关，并且通过抑制上皮间充质转化等途径起到抗肿瘤转移的作用，其阳性表达在非小细胞肺癌特别是肺腺癌中提示更好的预后。然而，在SCLC中，TTF-1的表达状况与预后关系的研究较少，且结论不一。本研究旨在探讨TTF-1表达状态与SCLC的一线含铂化疗敏感性及预后的关系，以期为SCLC的个体化治疗提供一定见解。

## 1 资料与方法

### 1.1 一般资料

回顾性分析2017年1月1日-2019年1月1日期间在青岛大学附属医院确诊并治疗的SCLC患者。纳入标准：①在青岛大学附属医院病理科进行病理学诊断，并同时行免疫组化TTF-1检测；②在青岛大学附属医院接受过连续系统的以铂类为基础的一线化疗，治疗方案包括依托泊苷联合卡铂/顺铂（EP/CE）、伊立替康联合卡铂/顺铂（IP/IC），至少进行2个周期化疗；③患者随访资料完整，依从性好。搜集患者的临床特征，包括年龄、性别、诊断日期、吸烟史、卡氏身体功能状态评分（Karnofsky performance status, KPS）、TTF-1表达情况、每2个周期化疗后的计算机断层扫描（computed tomography, CT）资料、一线治疗进展时间及死亡时间。共搜集了符合标准的患者234例，男177例（177/234, 75.6%），女57例（57/234, 24.4%）；年龄31岁-79岁，中位年龄60岁。根据美国退伍军人管理局肺癌研究组（Veterans Administration Lung Cancer Study Group, VALG）分期标准进行分期，局限期SCLC（limited-stage SCLC, LS-SCLC）133例（133/234, 56.8%），广泛期SCLC（extensive-stage SCLC, ES-SCLC）101例（101/234, 43.2%）；接受胸部放疗者139例（139/234, 59.4%），未行胸部放疗者95例（95/234, 40.6%）。

### 1.2 研究方法

#### 1.2.1 免疫组化结果判定标准

采用免疫组化EnVision两步法检测SCLC石蜡包埋组织中TTF-1表达，使用的检测试剂为TTF-1（D2E8）兔单克隆抗体#12373。免疫组化切片由本院两位经验丰富的病理科医生阅片审核，细胞核出现背景清晰的黄色或棕黄色颗粒为阳性反应。综合分析整张切片的阳性细胞数及着色强度，并将其记录为一个二元变量（阳性：所检测的任何肿瘤切片中的任何阳性反应；阴性：无反应）。

#### 1.2.2 观察指标

主要观察指标为一线化疗客观反应率（objective response rate, ORR）、疾病控制率（disease control rate, DCR）、无进展生存期（progression free survival, PFS）、总生存期（overall survival, OS）。根据实体瘤疗效评价标准（Response Evaluation Criteria in Solid Tumor, RECIST）1.1版评价疗效，分为完全缓解（complete response, CR）、部分缓解（partial response, PR）、稳定（stable disease, SD）和进展（progressive disease, PD）。OS指从确诊至患者死亡或者末次随访时间。PFS指一线治疗开始至疾病进展或者患者死亡的时间。ORR=（CR+PR）病例数/总病例数。DCR=（CR+PR+SD）病例数/总病例数。

#### 1.2.3 随访

采取病历资料查询或电话进行随访，随访截止至2019年12月1日，无失访患者。

### 1.3 统计学方法

采用SPSS 25.0进行统计学分析。TTF-1表达状态与临床特征及化疗反应率的关系采用*χ*^2^检验或*Fisher*确切概率法。ORR的多因素分析采用二元*Logistic*回归分析。采用*Kaplan-Meier*法进行患者生存单因素分析，生存率的比较采用*Log-rank*法进行显著性检验，采用*Cox*比例风险回归模型进行多因素生存分析。*P* < 0.05为差异有统计学意义。

## 2 结果

### 2.1 TTF-1的表达与临床特征的关系

234例SCLC患者中，根据免疫组化结果，TTF-1表达阳性为188例（80.3%, 188/234），TTF-1表达阴性为46例（19.7%, 46/234）。表 1显示，TTF-1表达状态与性别、年龄、有无吸烟史、KPS评分及分期均无关联（*P* > 0.05）。

**1:** 234例SCLC患者TTF-1表达与临床特征的关系[*n*（%）] Relationship between TTF-1 expression and clinical features in 234 patients with SCLC [*n* (%)]

Clinical features	*n*	TTF-1 (-)	TTF-1 (+)	*χ*^2^	*P*
Gender				0.213	0.644
Male	177 (75.6)	36 (78.3)	141 (75.0)		
Female	57 (24.4)	10 (21.7)	47 (25.0)		
Age (yr)				0.160	0.690
< 60	113 (48.3)	21 (45.7)	92 (48.9)		
≥60	121 (51.7)	25 (54.3)	96 (51.1)		
Smoking history				3.498	0.061
(-)	61 (26.1)	7 (15.2)	54 (28.7)		
(+)	173 (73.9)	39 (84.8)	134 (71.3)		
KPS score				3.543	0.060
≥80	164 (70.1)	27 (58.7)	137 (72.9)		
< 80	70 (29.9)	19 (41.3)	51 (27.1)		
Stage				1.895	0.169
LS	133 (56.8)	22 (47.8)	111 (59.0)		
ES	101 (43.2)	24 (52.2)	77 (41.0)		
Liver metastasis					0.113*
(-)	209 (89.3)	38 (82.6)	171 (91.0)		
(+)	25 (10.7)	8 (17.4)	17 (9.0)		
Brain metastasis					0.036*
(-)	220 (94.0)	40 (87.0)	180 (95.7)		
(+)	14 (6.0)	6 (13.0)	8 (4.3)		
Bone metastasis					0.180*
(-)	210 (89.7)	44 (95.7)	166 (88.3)		
(+)	24 (10.3)	2 (4.3)	22 (11.7)		
*Fisher exact probability method. KPS: Karnofsky performance status; LS: limited-stage; ES: extensive-stage; TTF-1: thyroid transcription factor-1; SCLC: small cell lung cancer.					

### 2.2 TTF-1的表达与化疗反应率的关系

表 2显示，TTF-1阳性患者一线化疗DCR为97.9%（184/188），高于TTF-1阴性患者的89.1%（41/46）（*P*=0.016）；TTF-1阳性患者一线化疗ORR为70.7%（133/188）高于TTF-1阴性患者的47.8%（22/46）（*χ*^2^=8.681, *P*=0.003）。分层分析显示，LS-SCLC患者中，TTF-1阳性表达者的ORR（74.8%）优于TTF-1阴性表达者（44.5%），差异有统计学意义（*χ*^2^=7.506, *P*=0.006）；ES-SCLC患者的ORR与TTF-1表达状态无关（*χ*^2^=1.722, *P*=0.189）。对化疗ORR进行*Logistic*多因素回归分析，结果如表 3所示，TTF-1表达是一线化疗ORR的独立预测因素（OR=0.395, 95%CI: 0.201-0.799, *P*=0.007），TTF-1阴性表达者一线化疗有效的可能性为阳性表达者的0.395倍。进一步将234例患者分成LS-SCLC亚组和ES-SCLC亚组，分别对两个亚组的一线化疗ORR进行*Logistic*多因素分析，结果显示LS-SCLC亚组中，TTF-1的阳性表达为患者ORR的独立预测因素（HR=0.265, *P*=0.008）；而ES-SCLC亚组中，TTF-1的表达状态与ORR无关（*P*=0.289）。

**2:** 234例SCLC患者TTF-1表达状态与化疗反应的关系[*n*（%）] Relationship between TTF-1 expression and chemotherapy response in 234 patients with SCLC [*n* (%)]

Items	TTF-1 (-)	TTF-1 (+)	*χ*^2^	*P*
CR	1 (2.2)	5 (2.7)	-	-
PR	21 (45.7)	128 (68.1)	-	-
SD	19 (41.3)	51 (27.1)	-	-
PD	5 (10.9)	4 (2.1)	-	-
DCR	41/46 (89.1)	184/188 (97.9)	-	0.016
ORR (total)	22/46 (47.8)	133/188 (70.7)	8.681	0.003
ORR (LS)	10/22 (45.5)	83/111 (74.8)	7.506	0.006
ORR (ES)	12/24 (50.0)	50/77 (64.9)	1.722	0.189
CR: complete response; PR: partial response; SD: stable disease; PD: progressive disease; ORR: objective response rate.				

**3:** 234例SCLC患者一线化疗ORR相关因素*Logistic*回归多因素分析 *Logistic* regression analysis of factors related to objective ORR of first-line chemotherapy in 234 patients with SCLC

Clinical features	*β*	*OR*	95%CI	*P*
Gender (Male *vs* Female）	0.257	1.293	0.644-2.594	0.470
Age (< 60 yr *vs* ≥60 yr)	-0.085	0.918	0.521-1.619	0.768
Stage (LS *vs* ES)	0.311	1.365	0.756-2.464	0.303
KPS score (≥80 *vs* < 80)	0.561	1.753	0.949-3.237	0.073
Smoking history (- *vs* +)	-0.265	0.767	0.385-1.529	0.451
TTF-1 (- *vs* +)	-0.928	0.395	0.201-0.799	0.007

### 2.3 TTF-1的表达与SCLC预后的关系

图 1显示，234例SCLC患者中，TTF-1阳性者的中位PFS为9.0个月，长于TTF-1阴性者的6.9个月（*χ*^2^=9.357, *P*=0.002）。TTF-1阴性者的中位OS为13.3个月，短于TTF-1阳性者的20.1个月（*χ*^2^=12.082, *P*=0.001）。表 4显示，对性别、年龄、吸烟史、KPS评分、TTF-1表达、是否行胸部放疗及分期进行单因素预后分析，发现有无吸烟史（*χ*^2^=4.015, *P*=0.045）、KPS评分（*χ*^2^=48.503, *P* < 0.001）、TTF-1表达（*χ*^2^=12.082, *P*=0.001）、是否行胸部放疗（*χ*^2^=16.600, *P* < 0.001）及分期（*χ*^2^=25.015, *P* < 0.001）是影响OS的重要因素。进一步进行*Cox*回归多因素生存分析，发现分期（HR=0.602, 95%CI: 0.447-0.810, *P*=0.001）、KPS评分（HR=0.368, 95%CI: 0.267-0.506, *P* < 0.001）、TTF-1表达（HR=1.724, 95%CI: 1.210-2.455, *P*=0.003）、是否行胸部放疗（HR=1.455, 95%CI: 1.087-1.949, *P*=0.012）为OS的独立预后因素。TTF-1阳性、KPS评分≥80分、局限期、行胸部放疗的患者OS分别优于TTF-1阴性、KPS评分 < 80分、广泛期、未行胸部放疗的患者。进一步进行分层分析显示，LS-SCLC亚组中，TTF-1阴性表达患者的中位OS为16.5个月，TTF-1阳性表达患者的中位OS为20.5个月，差异有统计学意义（*P* < 0.001）；ES-SCLC亚组中，TTF-1阴性表达患者的中位OS为12.1个月，TTF-1阳性表达患者的中位OS为14.9个月，差异有统计学意义（*P*=0.001）。TTF-1表达状态为局限期以及广泛期SCLC患者OS的独立预后因素。

**1:**
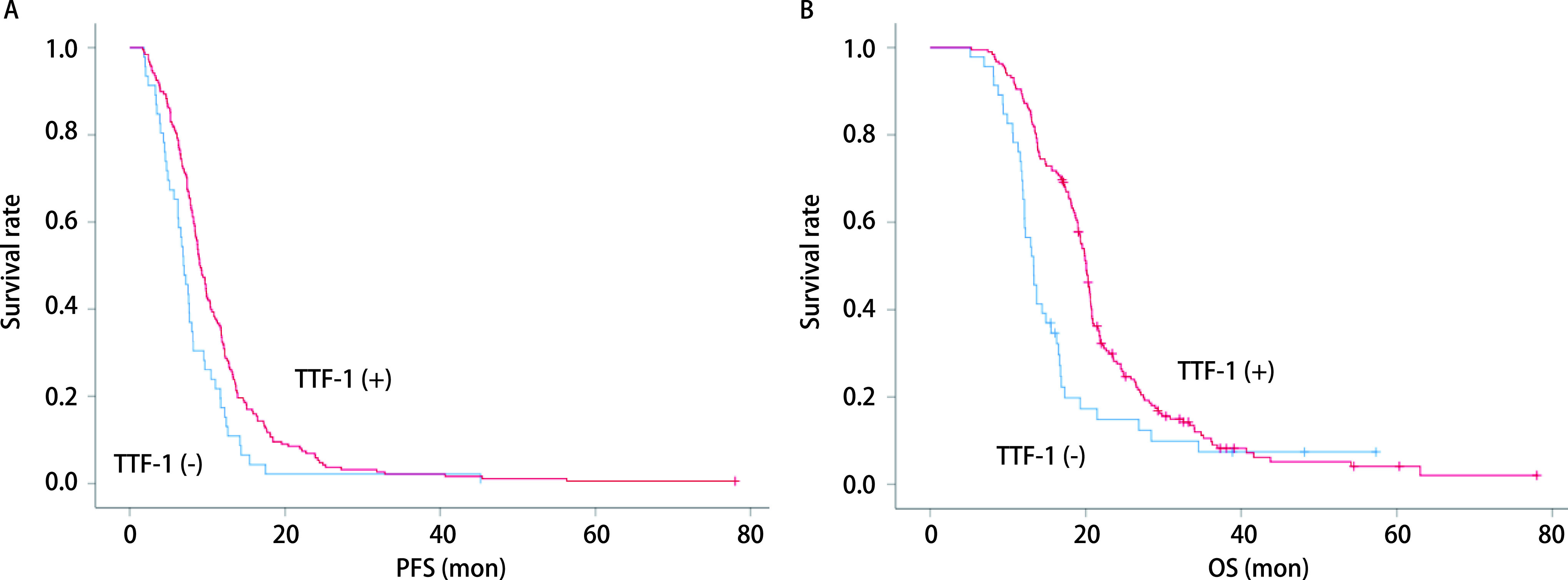
生存曲线。A：234例SCLC患者TTF-1表达与PFS曲线（*P*=0.002），TTF-1（-）中位PFS 6.9个月，TTF-1（+）PFS 9.0个月；B：234例SCLC患者TTF-1表达与OS曲线（*P*=0.001），TTF-1（-）中位OS 13.3个月，TTF-1（+）OS 20.1个月。 Survival curve. A: *Kaplan*-*Meier* survival curves of patients with SCLC. Comparison between TTF-1 positive and TTF-1 negative groups shows a tendency for better PFS in the TTF-1 positive group (*Log*-*rank* test: *P*=0.002); B: *Kaplan*-*Meier* survival curves of patients with SCLC. Comparison between TTF-1 positive and TTF-1 negative groups shows a tendency for better cumulative survival in the TTF-1 positive group (*Log*-*rank* test: *P*=0.001). PFS: progression free survival.

**4:** 234例患者临床病理特征与OS关系的单因素及*Cox*回归多因素分析 Univariate and *Cox* regression multivariate analysis of the relationship between clinicopathological features and OS in 234 patients

Clinical features	Univariate survival analysis			Multivariate survival analysis	
	*χ*^2^	*P*		HR (95%CI)	*P*
Gender (Male *vs* Female)	2.565	0.109			
Age (< 60 yr *vs* ≥60 yr)	0.702	0.402			
Smoking history (- *vs* +)	4.015	0.045		0.793 (0.577-1.091)	0.154
Stage (LS *vs* ES)	25.015	< 0.001		0.602 (0.447-0.810)	0.001
KPS score (≥80 *vs* < 80)	48.503	< 0.001		0.368 (0.267-0.506)	< 0.001
TTF-1 (- *vs* +)	12.082	0.001		1.724 (1.210-2.455)	0.003
Chest radiotherapy (- *vs* +)	16.600	< 0.001		1.455 (1.087-1.949)	0.012
OS: overall survival.					

**5:** LS-SCLC和ES-SCLC的*Cox*多因素分析 *Cox* multivariate analysis in patients with LS-SCLC or ES-SCLC respectively

Clinical features	LS-SCLC			ES-SCLC	
	HR (95%CI)	*P*		HR (95%CI)	*P*
Gender (Male *vs* Female）	0.864 (0.538-1.39)	0.548		0.933 (0.523-1.664)	0.814
Age (< 60 yr *vs* ≥60 yr)	0.806 (0.546-1.188)	0.275		1.143 (0.739-1.767)	0.548
Smoking history (- *vs* +)	0.82 (0.527-1.276)	0.378		0.708 (0.378-1.325)	0.280
Chest radiotherapy (- *vs* +)	1.566 (1.008-2.433)	0.046		1.528 (0.969-2.411)	0.068
KPS score (≥80 *vs* < 80)	0.309 (0.194-0.493)	< 0.001		0.478 (0.301-0.759)	0.002
TTF-1 (- *vs* +)	1.622 (1.264-2.018)	< 0.001		2.289 (1.400-3.743)	0.001

## 3 讨论

TTF-1作为肿瘤神经内分泌分化过程中的关键调节因子，在SCLC中高表达，既往研究^[8]^指出其在SCLC中的表达阳性率高达80%-90%，我们的研究中，234例SCLC患者TTF-1阳性率为80.3%，与既往报道基本一致，且TTF-1的表达状态与性别、年龄、吸烟史、KPS评分及肿瘤分期、初诊时有无肝转移及骨转移等均无关。TTF-1阴性患者比阳性患者初诊时有更高的脑转移可能性（13.0% *vs* 4.3%, *P*=0.036），这提示TTF-1阴性的SCLC可能具有更强的侵袭转移能力，这在肺腺癌中已被证实^[9-11]^。

与非小细胞肺癌相比，SCLC患者一线化疗的有效率较高。然而，仍然有10%-20%的SCLC患者对一线化疗没有反应，这其中的原因是未知的。众所周知，化疗会引起严重的副作用，降低患者的生活质量，因此有必要确定可以预测化疗反应的标志物。在我们的研究中，234例SCLC患者中，TTF-1表达阳性组患者一线化疗DCR为97.9%，ORR为70.7%；阴性组分别为89.1%、47.8%（*P* < 0.05），可见TTF-1阳性组对化疗的反应优于TTF-1阴性组，这提示TTF-1有可能成为一线化疗反应的预测指标。这与国内外的某些研究^[12, 13]^结果一致。然而，我们的研究中发现，这种差异主要体现在局限期，广泛期患者TTF-1表达状态与化疗ORR之间未见显著相关性，且TTF-1阴性亚组的患者疾病控制率仍然较高，因此，就我们的研究来说，仅使用TTF-1表达状态作为铂类化疗获益的患者的筛选标准是不足的。TTF-1与其他生物标志物联合使用是否具有预测价值，有待进一步研究。然而我们的研究中，并未涉及TTF-1表达状态与化疗药物的毒副反应之间是否存在一定的关系。

既往研究报道了多种SCLC的预后指标，如性别^[14]^、有无吸烟史^[15]^、相关血液学指标^[16-19]^、体能状态评分^[20]^及不同的治疗策略^[21]^。在我们的研究中，肿瘤分期、KPS评分、TTF-1表达状态及是否行胸部放疗是SCLC的独立预后因素。既往关于TTF-1表达状态在SCLC中的预后作用研究结果相互矛盾。Yan等^[22]^研究认为TTF-1表达阳性的SCLC患者的PFS和OS较差。Misch等^[13]^对221例SCLC患者的生存分析发现，不同TTF-1表达的患者之间的比较没有显著差异，TTF-1在SCLC患者中的表达无预后意义。在一纳入11项研究、涉及1, 786例SCLC患者的*meta*分析^[23]^中指出TTF-1表达阳性的患者OS和PFS较阴性患者有所延长。本研究结果显示，TTF-1阳性表达组与阴性表达组患者的PFS及OS差异有统计学意义，TTF-1阴性亚组患者的预后比阳性组更差，TTF-1阴性表达预示着更高的死亡风险。出现这些不同结果的原因可能为虽然所有研究都使用免疫组织化学方法检测TTF-1的表达状况，但它们所使用的阳性标准可能有所不同，这可能是一个混杂因素。同样，患者群体之间也存在差异，且目前包括我们的研究在内的大多数研究样本量较少，且均为回顾性研究。

综上所述，我们的研究表明TTF-1可能成为一种新的SCLC化疗敏感性和生存预后的生物标志物。然而，确切的结论需要更大样本量的前瞻性研究及SCLC发生发展过程中的分子机制研究加以证实。
